# Enhanced YOLOv8n-Based Three-Module Lightweight Helmet Detection System

**DOI:** 10.3390/s25247664

**Published:** 2025-12-17

**Authors:** Xinyu Zuo, Yiqing Dai, Chao Yu, Wang Gang

**Affiliations:** 1 School of Architecture and Design, China University of Mining and Technology, Xuzhou 221000, China; 18796331435@163.com; 2 College of Civil Engineering, Fuzhou University, Fuzhou 350108, China; yuchao2580@126.com; 3 University of Chinese Academy of Sciences, Beijing 100049, China

**Keywords:** construction safety, YOLOv8n, helmet detection, lightweight

## Abstract

Maintaining a safe working environment for construction workers is critical to the improvement of urban areas. Several issues plague the present safety helmet detection technologies utilized on construction sites. Some of these issues include low accuracy, expensive deployment of edge devices, and complex backgrounds. To overcome these obstacles, this paper introduces a detection method that is both efficient and based on an improved version of YOLOv8n. Three components make up the superior algorithm: the C2f-SCConv architecture, the Partial Convolutional Detector (PCD), and Coordinate Attention (CA). Detection, redundancy reduction, and feature localization accuracy are all improved with coordinate attention. To further enhance feature quality, decrease computing cost, and make corrections more effective, a Partial Convolution detector is subsequently constructed. Feature refinement and feature representation are made more effective by using C2f-SCConv instead of the bottleneck C2f module. In comparison to its predecessor, the upgraded YOLOv8n is superior in every respect. It reduced model size by 2.21 MB, increased frame rate by 12.6 percent, decreased FLOPs by 49.9 percent, and had an average accuracy of 94.4 percent. This method is more efficient, quicker, and cheaper to set up on-site than conventional helmet-detection algorithms.

## 1. Introduction

The construction industry is a vital component of the national economy in the contemporary context of China’s rapid industrialization, rendering its development indispensable. This sector has traditionally been categorized as high-risk, owing to the widespread use of large-scale construction equipment, extensive operations at elevated heights, and significant labor attrition. Construction site safety issues negatively impact the daily lives of nearby residents and impede the standard operational schedules of the project, thereby causing substantial financial losses and compromising the rights and interests of individuals. In the construction industry, the most common causes of fatal and non-fatal accidents are falls from heights, being struck by objects, machinery-related injuries, collapses, and electrical shocks, according to statistical data. Falls from heights and impacts with objects constitute approximately 65% of all accidents [1]. Scientific evidence suggests that the proper use of safety helmets can significantly reduce the risk of severe head injuries during falls from heights and reduce impact injuries from falling objects. As a result, safety helmets are indispensable personal protective equipment (PPE) that safeguards the lives of construction laborers.

Wearing safety helmets is a key priority under current safety management regulations for construction sites, and safety violations carry severe penalties [2,3]. As practice shows, safety guidelines must be strictly enforced to prevent accidents. Many workers still do not understand the need for safety helmets in construction. Traditional safety supervision approaches use on-site safety personnel for manual patrols and oversight. This method has many practical difficulties. Large construction sites with complex, dynamic work conditions and substantial human mobility are common. Due to these factors, manual supervision cannot fully monitor all workers’ helmet-wearing status. Due to inefficient oversight, workers cannot quickly identify and address violations. Next, the safety officer at a construction site needs to monitor this area during working hours. Security personnel become fatigued because they must carefully observe the area for several hours, and the cost of construction inspection safety increases. This makes ensuring safety and detecting safety violations over a long period unsuitable. The widespread presence of workplaces across the country exacerbates these issues, disrupts safety regulations, and often makes it difficult to attribute safety responsibilities, with no one knowing who is at fault in terms of safety.

With the development of machine vision and artificial intelligence, technologies can help intelligent machines identify safety issues, which can improve the safety of construction sites. The Eye Spy helmet system uses a small computer and many cameras, which can guard the construction site 24 h a day, which is different from the old method. It manages safety rules through online evaluation and improves accuracy. This technology can find out whether the workers are wearing safety helmets, which helps to establish long-term safety rules, help to make management plans, and also allows security officers to find problems quickly. Later, models such as Faster R-CNN, YOLO series and RetinaNet became more and more powerful, allowing people to study more carefully how to detect the wearing of helmets [4]. However, the actual situation of the construction site is very complicated; the light may change, something may block it, and the surrounding environment may be messy. A core goal of this technology is to improve its real-time processing efficiency and simplify the object detection algorithm without affecting the accuracy. In this way, the detection system will be more stable and easier to adapt to different working environments.

**Figure 1:**
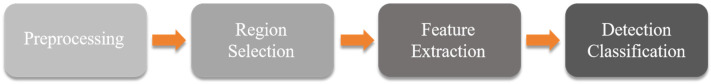
The steps of the traditional object detection algorithm.

Figure 1 shows the usual workflow of traditional object detection algorithms. It can be broken down into four steps: preprocessing, generating region proposals, extracting features, and classifying and regressing. This diversity-based method generates a lot of possible bounding boxes in the picture to frame the candidate areas.

The development of deep learning has promoted great progress in computer vision, especially in object recognition. Scholars have deeply studied the adaptation of deep learning to identify safety helmets. However, the application in practical engineering scenarios still faces challenges. Finding a balance between detection accuracy and calculation requirements has always presented a difficult problem. This shows that the complexity of the environment directly affects the performance, especially on construction sites, where factors such as large light changes, workers being blocked, and various colors of helmets increase the risk of misjudgment and affect the reliability of the system [5]. In addition, limited processing resources also hinder field deployment. Although deep learning performs well in detection tasks, many models require a lot of computing resources [6]. The demand for high-performance hardware increases costs, and it cannot be used in small portable devices.

Existing solutions have found that it is difficult to conduct intelligent security checks within construction sites, mainly due to the complexity of the scenes and the limitations of keeping the model lightweight and easy to use, which hinders the progress of the work [7]. The main objective of this study is to adjust the running mode of the model to have a lower computing cost but still enable accurate detection on edge devices.

This study introduces an innovative method (YOLOv8n-L) that utilizes the architecture of YOLOv8n to enhance the algorithm structure for detection, reduce computational workload, and solve the detection problem of existing algorithms. By improving the algorithm and solving the safety problems on the construction site, especially using YOLOv8n to quickly and accurately detect and set up small equipment, this method can come in handy.

We propose a more convenient algorithm, YOLOv8n-L, to detect helmets, enhance functionality, and solve the problem of YOLOv8n inspecting helmets and detecting small target sizes on construction sites. Introducing a coordinated attention coordinate mechanism in the main network can better detect and highlight features in complex scenes. Then, we designed a lightweight Partial Convolution detector head that uses Partial Convolution to keep the model small and enable fast detection. We constructed a C2f SCConv module by modifying the C2f structure. This ensures the sensitivity of detection and promotes the detection effectiveness of lightweight network design. These adjustments help to discover targets of different sizes, especially tiny ones, and truly magnify the features of the safety helmet. Tests have shown that the enhanced model greatly improves detection performance in complex building scenes. After improvement, the YOLOv8n-L model could more easily detect safety on construction sites. Compared with the original YOLOv8n algorithm, the new YOLOv8n-L performs better in detecting things, and it is faster and easier to deploy. It has become easy to achieve the real-time detection of targets on small devices.

This article has adjusted the internal architecture of the model, finding a balance between detection efficiency and not requiring a large amount of computer power. After some adjustments, the YOLOv8n-L model performs well in small devices and edge computing settings. It can accurately detect the wearing of safety helmets and lightly mark them. Compared to previous detection and method techniques, it is much better in terms of the flexible use of computer power, real-time detection, and flexible deployment. This modification provides a reliable and effective technical method that can meet the needs for intelligent safety monitoring on construction sites. We achieved the following in this paper:

1. A lightweight architecture utilizing coordinate attention (CA) was developed to enhance long-range spatial modeling and decrease deployment computational costs.
2. A lightweight decoupled detection head was constructed utilizing a Partial Convolution detector (PCD). This minimizes redundant convolution operations and facilitates extraction from complex and dynamic architectural environments.
3. The C2f-SCConv structural module systematically substitutes standard bottleneck modules with SC_bottleneck path units, optimizing feature modeling and improving the learning capacity of cross-level feature diversity across spatial dimensions.

The rest of the study is set up as follows: In Section 2, the literature review analyzes changes in the time of deep learning through the study of literature, and summarizes the gaps and deficiencies in the study. Section 3 explains how the YOLOv8n organizational structure and the advanced improved YOLOv8n-L structure work, as well as three key parts: coordinate attention (CA), Partial Convolution Detector (PCD) and C2f-SCConv. How the three modules make the model better is explained in detail. In Section 4, we discuss the experimental setup, datasets, comparative experiments, and ablation studies. Section 5 summarizes the achievements of the preceding sections, analyzes limitations, and proposes future research directions. Section 6 concludes our experimental work.

## 2. Related Research

The widespread use of deep learning and its rapid advances in computer vision have led to significant improvements in object detection algorithms for complex recognition tasks. This new technology gives us a stronger, more creative way to detect whether someone is wearing a hard hat automatically. As the need for improved performance from safety helmet detection systems at construction sites grows, research is increasingly focused on deep network optimization and better feature integration across different scales. This is especially true for high-precision recognition, low-latency real-time response, and adaptability to resource-constrained environments. The goal of these changes is to better meet the needs of real-world use.

Researchers have made many algorithmic improvements in the last few years. For instance, Waranusast et al. [8] developed a KNN classifier that uses features from moving objects to determine whether someone is wearing a helmet, primarily for motorcycle riders. Nevertheless, their methodology relied on extracting head-region features, which reduced accuracy when applied to low-resolution images and overlapping objects.

Fang et al. [9] developed a system that utilized an R-CNN-based approach to confirm that construction laborers were wearing helmets as required. They randomly selected 100,000 frames of construction worker images from surveillance recordings at 25 distinct construction sites. The data covered 1 year and showed an increase in efficacy. Sridhar et al. [10] implemented the YOLO v2 deep learning framework to identify helmet utilization among cyclists. They developed a more effective approach by using a single convolutional neural network (CNN) to classify helmet status for motorcyclists. The proposed YOLO v2 architecture outperformed conventional algorithms in experiments. Nipun et al. [11] proposed detecting PPE compliance using a deep learning model based on YOLOv3. Using a single CNN framework, this algorithm verifies PPE compliance and detects individual workers. All models were trained on an internal image dataset generated through crowdsourcing and web mining to achieve exceptional outcomes and advance construction automation research.

Ben et al. [12] implemented a modified YOLOv4 algorithm for helmet detection to supervise the utilization of safety helmets by employees in production environments. They collected and integrated datasets and then clustered prior frame sizes using the K-means algorithm. Implementing a multi-scale training strategy improved the model’s performance across scales, yielding exceptional results. Gao et al. [13] presented the multi-coupled, multi-scale small-object detection algorithm, DMS-YOLOv5. Adding a perception module makes it easier to focus on low-resolution targets. At the same time, a coordinate attention mechanism was implemented that combines channel and spatial attention data. This setup helps the system focus on what’s important and ignore the random stuff, boosting how well it finds key details and does its job.

Sun et al. [14] shared a simple way to spot helmets using YOLOv5. For better and more quickly spotting helmets in areas where buildings are going up, they put a special focus tool called MCA into the main system. It became better at seeing safety helmets on workers, missed fewer small helmets, and worked faster too.

Shao et al. [15] added coordinate attention and MBConv modules to YOLOv7, which can better detect small targets. At the same time, they also improved the object positioning technology to make the overall efficiency higher.

Yung et al. [16] evaluated the ability of YOLOv5, YOLOv6 and YOLOv7 models in identifying the use of protective equipment. In order to see how effective these algorithms are in detecting safety helmets, they conducted a series of tests and then analyzed and compared the results.

Jung et al. [17] conducted a comparative performance test using YOLOv7 and YOLOv8 to generate defect images of vehicle body parts via GANs. The experimental results demonstrate that YOLOv8 outperforms YOLOv7 due to its faster processing speed and higher detection efficiency.

Fan et al. [18] built a feature-enhanced YOLOv8 detection tool that integrates feature maps using BiFPN while reducing model size. This enables it to better capture helmet features, thereby assisting detection tasks with reduced computational demands. Consequently, this algorithm significantly enhances the effectiveness of related detection work.

Luo et al. [19] made an upgraded version of YOLOv8 to identify safety devices. They changed the input part of YOLOv8 so that the model became smaller and the training speed was faster. These changes are to make the algorithm better and more accurate.

According to the previous studies, we can sum up some key points. Conventional algorithms do not work for construction environments right now because they are too complicated to compute and take too long to make decisions. These methods are not practical on edge devices with limited resources because they require significant processing power. If you want better real-time monitoring, look for a lightweight detection model that can make inferences faster. But they do not work as well in complex, constantly changing field conditions, such as when there are many obstructions and changes in lighting. Helmet detection technology still faces problems, even though the field has made significant progress. This is because construction sites need fast, intelligent monitoring and lightweight edge deployment. Reduced identification accuracy is evident in scenarios characterized by minor object occlusions, intense illumination, diverse helmet appearances, and congested environments [20]. In this research, we utilize an improved YOLOv8n architecture for helmet detection. A Coordinated Attention method makes it easier for the model to focus on critical areas. To streamline the calculation, a Partial Convolution Detector is used to separate the head. There has been an upgrade to feature extraction and transmission with the new C2f-SCConv module. This technology improves complex scenario management, algorithmic lightness, inference efficiency, and helmet detection for small targets and blocked objects.

## 3. Optimization Mode of the YOLOv8n Algorithm

### 3.1. YOLOv8n Network

**Figure 2:**
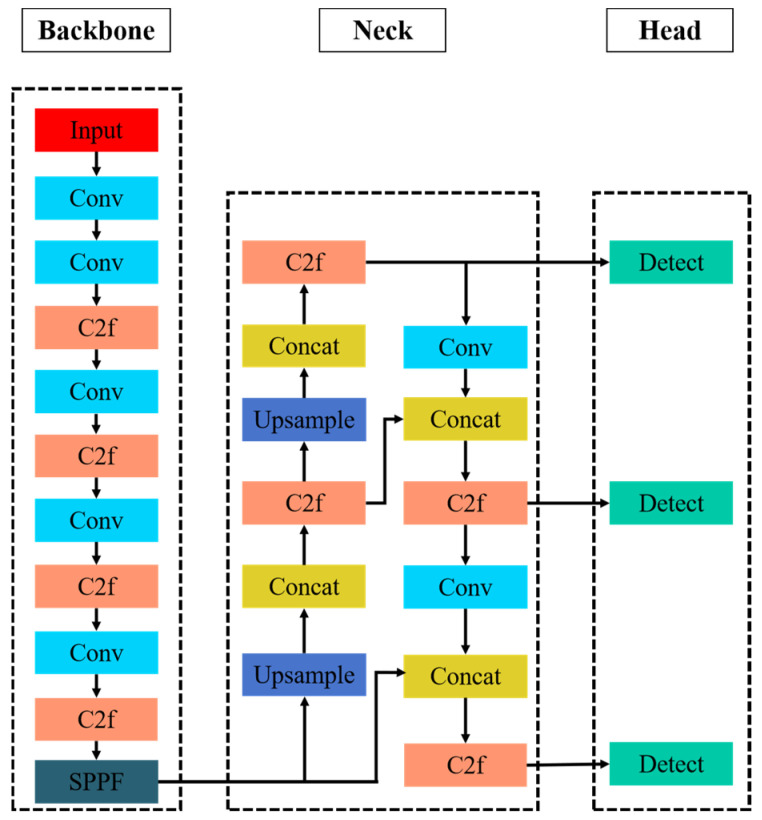
The design of YOLOv8n.

Scalability and setup flexibility are YOLOv8’s primary advantages over its predecessors. This model preserves backward compatibility with existing YOLO designs, allows users to choose between model versions based on specific requirements, and enables them to freely alter model structures and hyperparameter settings. One of numerous YOLOv8 variations is YOLOv8n. YOLOv8n was selected for detection due to its modest size and high accuracy, given model size and computational complexity. The YOLOv8n network architecture is depicted in Figure 2 and consists of an input layer, an output layer, a neck network, a backbone network, and submodules, along with their connections [21].

1. Input: Preprocessing and augmenting helmet-wearing photos improve the model’s flexibility to different settings.
2. Backbone: The backbone optimizes structures using CSP (Convolutional Self-Projection). CBS, C2f, and SPPF modules make up most of it. YOLOv8 received the SPPF module from YOLOv5. This Spatial Pyramid Pooling (SPPF) module transforms feature maps of varying sizes into uniformly sized feature vectors, thereby fusing local and global information for better feature representation [22].
3. Neck: The network utilizes multi-scale features derived from the backbone to improve the model’s ability to detect objects of different sizes. It maintains the FPN and PAN architecture to enhance multi-scale feature interaction, thereby constructing a feature pyramid that transmits semantic and localization information across layers.
4. Head: A head that is not connected to the body replaces the head that is attached. Separate network branches for each of the main subtasks of object detection—classification and bounding box regression—are an improvement [23]. This method of separating tasks reduces conflicts between classification and localization during feature learning, which leads to task-specific representations. We stopped using IoU-based matching and fixed-ratio single-sample allocation methods to divide positive and negative samples. Instead, we use the Task-Aligned Assigner. The Intersection over Union (IoU) between predicted and ground-truth boxes, along with the classification confidence score, is used to dynamically assign the correct labels and regression targets to each anchor or predicted box. This improved, flexible allocation method makes it easier to identify and select high-quality, informative samples, while reducing the impact of low-quality or confusing data. As a result, it dramatically improves sample matching stability.

### 3.2. YOLOv8n-L Architecture Network

**Figure 3:**
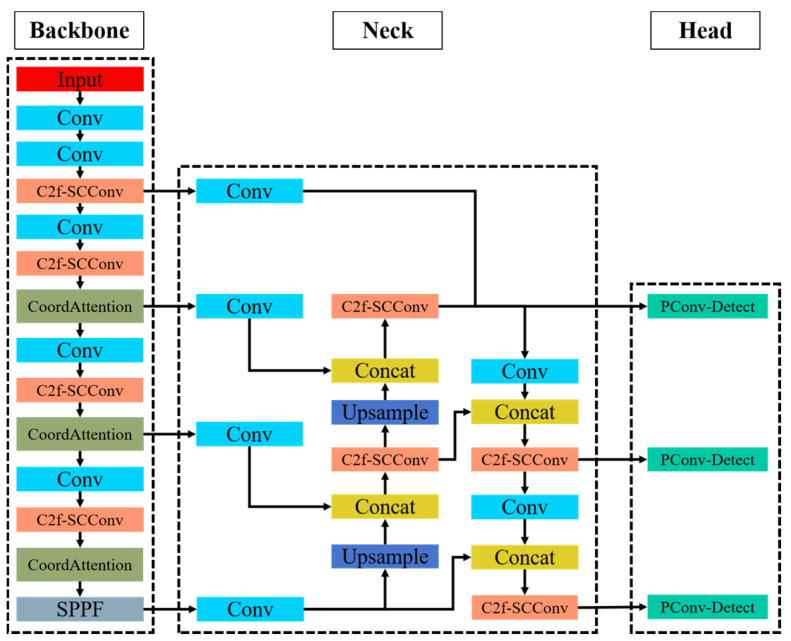
The architecture of YOLOv8n-L.

The research plan of this paper makes some changes to solve the main problems encountered by the YOLOv8n model in finding helmets, such as cluttered background, small target size and objects blocking the line of sight, which makes it less correct and difficult to detect. By using the methods of improving the model structure, lightening the model size and making the function, the working efficiency of the model is truly improved. Here are some specific ways to optimize it:

1. Putting CoordAttention into the backbone network after the C2f module. This method accurately maps the spatial distribution of areas where people wear helmets by decomposing spatial coordinates to simulate channel linkages and flexibly model long-range spatial dependencies. This architecture significantly improves the model’s ability to focus on target areas by reducing background noise, thereby enhancing feature discrimination and localization accuracy, even in high-noise environments.
2. We suggest replacing the coupled detection structure in the original YOLOv8n with PConv-Detect, a lightweight decoupled detection head based on Partial Convolution. This will make the model simpler and speed up computation. This architecture reduces the number of parameters and the computational cost, and eliminates task-specific optimization. It also makes resource allocation easier by separating classification and localization.
3. The C2f-SCConv module is developed by incorporating Spatial and Channel Reconstruction Convolution (SCConv). During feature fusion, this module employs a dynamic redundancy-compression mechanism to select high-contribution features and suppress low-information redundant features autonomously. Its benefits include reduced redundant computations, fewer parameters, greater robustness to small objects and occlusions, and stronger feature extraction for local details and occluded objects. Figure 3 illustrates the enhanced YOLOv8n-L network architecture.

### 3.3. Coordinate Attention

Detecting safety helmets is a classic challenge because they typically occupy a small pixel area in images and are susceptible to interference from complex background textures and uneven illumination conditions [24]. This paper adds an attention module to the YOLOv8n backbone to improve recognition reliability and the model’s ability to evaluate essential head areas. By dynamically adjusting the spatial weights of feature maps, this design significantly enhances its ability to capture spatial information about safety helmets.

**Figure 4:**
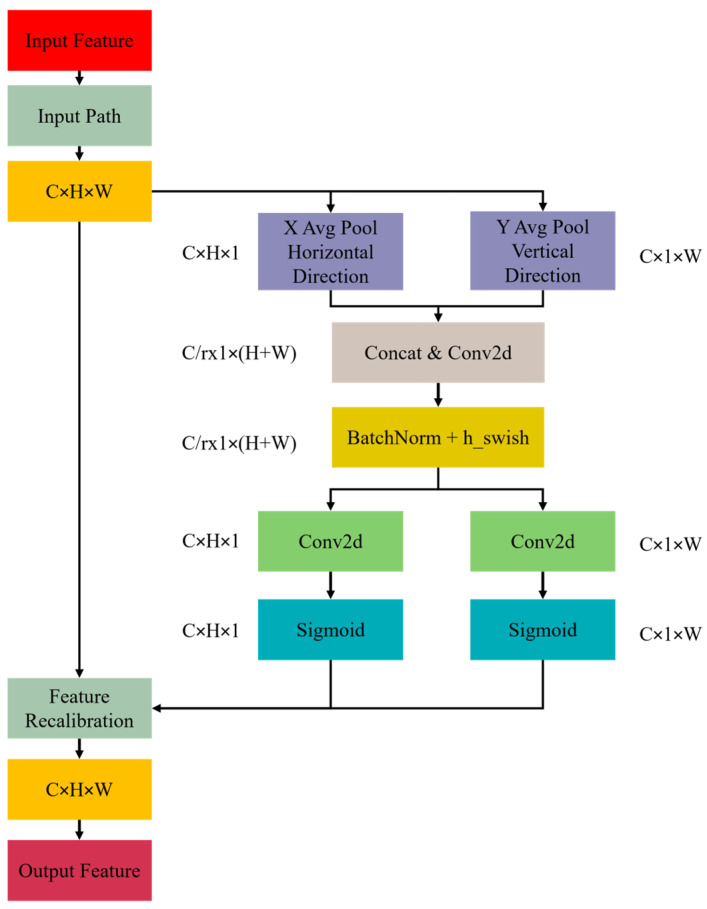
The structure of the attention mechanism.

Figure 4 shows the structure of the attention mechanism, which enables the model to focus on important information while filtering out unnecessary features. This makes the model better at detecting things overall. Coordinated attention is like a little guide, enhancing the model’s focus on key points, so it is better at detecting and labeling what it sees.

Coordinated attention is a very simple mechanism, which can make the model "see" the picture more clearly by guiding the model to pay attention to the important places and marking them well. If there is a picture with the size of C×H×W, its features will be displayed in two different ways: (H, 1) from top to bottom and (1, W) from left to right. Equations (1) and (2) show the principle. Formula (1) obtains the large feature score of channel *c* at height *h* by averaging each pixel value on the width at height *h*. The width of the mapping *Xc*(*h*, *i*) tells the value of the pixel at height *h*, width *i*, and channel *c*. As shown in Formula (2), the feature score of channel *c* at width *w* comes from averaging all height values at that width. *Xc*(*j*, *w*) is the pixel value at height *j*, width *w*, and channel *c*. H is the height of the feature map. The model efficiently encodes long-range dependencies along one spatial dimension while maintaining positional integrity in the other dimension. The decoded results from both directions are turned into feature maps that include information about direction and position. These maps are then used to add to the features of the original input.

(1) $$Z_{c}^{h}\left(h\right)=\frac{1}{w}\sum_{0\leq i<W}X_{c}\left(h,i\right)$$

(2) $$Z_{c}^{w}\left(w\right)=\frac{1}{H}\sum_{0\leq j<H}X_{c}\left(j,w\right)$$

### 3.4. Constructing Partial Convolution Detection

Partial Convolution changes how regular convolutions work, and it is the main component of the lightweight backbone network FasterNet, which makes models much simpler [25]. It proposes a more efficient method for convolution computation, performing standard convolution operations on a restricted subset of input channels to obtain crucial spatial information. An identity mapping then sends the residual channels. This architecture reduces computational redundancy and improves the efficiency of floating-point operations, enabling more effective processing without significantly compromising accuracy.

The initial and terminal channels of the input feature map constitute the Critical Feature Subset for convolution in this architecture. To develop a universal analysis model, assume that the input and output feature maps have the same number of channels. The FLOPs of Partial Convolution (PConv) are represented by Formula (3).

(3) $$FLOPs_{PConv}=h\times w\times k^{2}\times {c_{p}}^{2}$$

The height and width of the feature map are denoted as h and w, respectively; the convolution kernel is represented by *k*, and the number of channels involved in the convolution process is *c_p_*. When the partial channel ratio is 1/4, Partial Convolution FLOPs are about 1/16 of conventional convolution. The memory use of Partial Convolution is also calculated in Formula (4).

(4) $${mem}_{PConv}=h\times w\times 2c_{p}+k^{2}\times c_{p}^{2}\approx h\times w\times 2c_{p}$$

**Figure 5:**
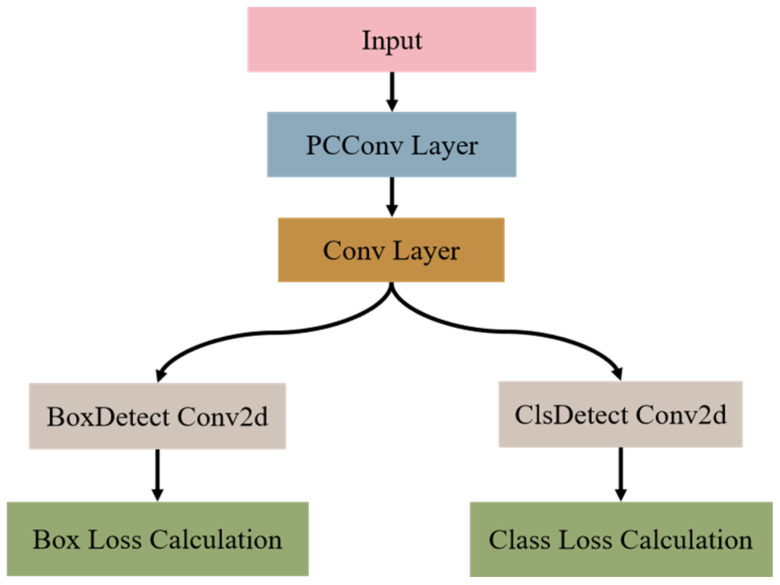
Structure of PConv-Detect.

This paper presents PConv-Detect, a lightweight, decoupled detection head that mitigates computational redundancy in the YOLOv8n model. The detection head now uses hierarchical feature processing rather than the original 4 3 × 3 convolutional layers. The structure has two cascaded heterogeneous convolutional units. First, the channel-selective spatial filtering layer uses a 3 × 3 PConv as its core operator. This suppresses redundant feature responses by sparse convolution of a continuous subset of input feature channels. Second, a 1 × 1 standard convolutional layer follows the channel-wise projection layer. It recalibrates feature channels using learnable weight matrices to simulate cross-channel correlations and improve feature discrimination. Combining these subunits optimizes feature representation for enhanced detection and reduced computational resources. The final version of the improved detector head structure is shown in Figure 5.

### 3.5. Construction of C2f-SCConv

Convolutional neural networks (CNNs) are widely used in computer vision applications; however, their significant computational and storage requirements pose implementation challenges. Research indicates that the intrinsic spatial and channel feature redundancy in standard convolutional layers constitutes a critical limiting factor. Even though traditional compression methods, such as network pruning and knowledge distillation, can improve performance, they both depend on the quality of pre-trained models [26]. Li et al. [27] proposed the Spatial-Channel Convolution (SCConv) module to address this problem. SCConv jointly optimizes feature spatial correlations and channel dependencies to remove redundant features. You can easily add this plug-and-play module to your existing CNN architectures to improve their efficiency [28].

**Figure 6:**
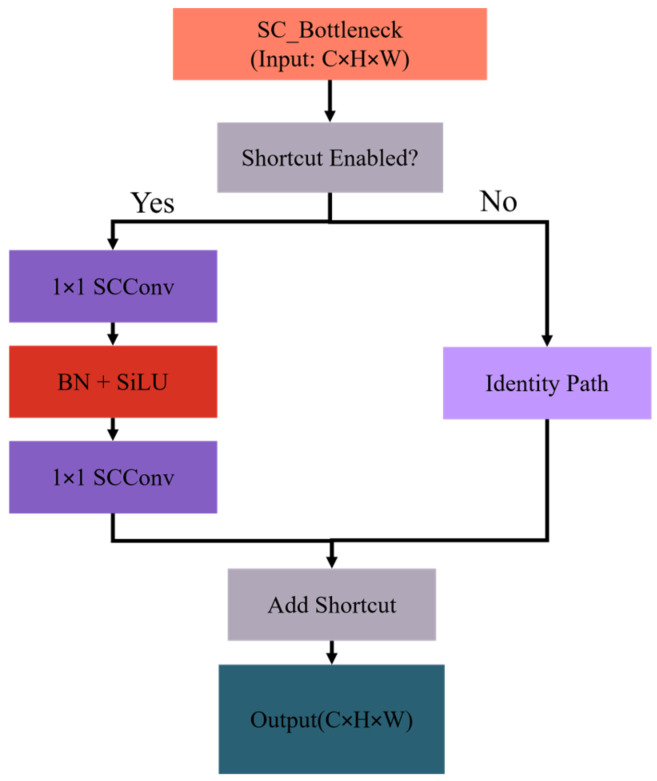
Structure of split-channel bottleneck.

This research presents SCConv, which restructures the C2f network architecture in YOLOv8n to alleviate the high model complexity and significant FLOPs associated with current safety helmet detection techniques. This method improves detection by eliminating unnecessary duplication in the network parameters. The SC_Bottleneck structure, as shown in Figure 6, serves as an expansion layer initially to add more feature channels. After batch normalization, the SiLU activation function is applied to the output to capture nonlinear relationships better. The second SCConv module subsequently reduces the number of channels in the feature map to match the input channel count. Ultimately, the output is produced by superimposing the input feature map.

**Figure 7:**
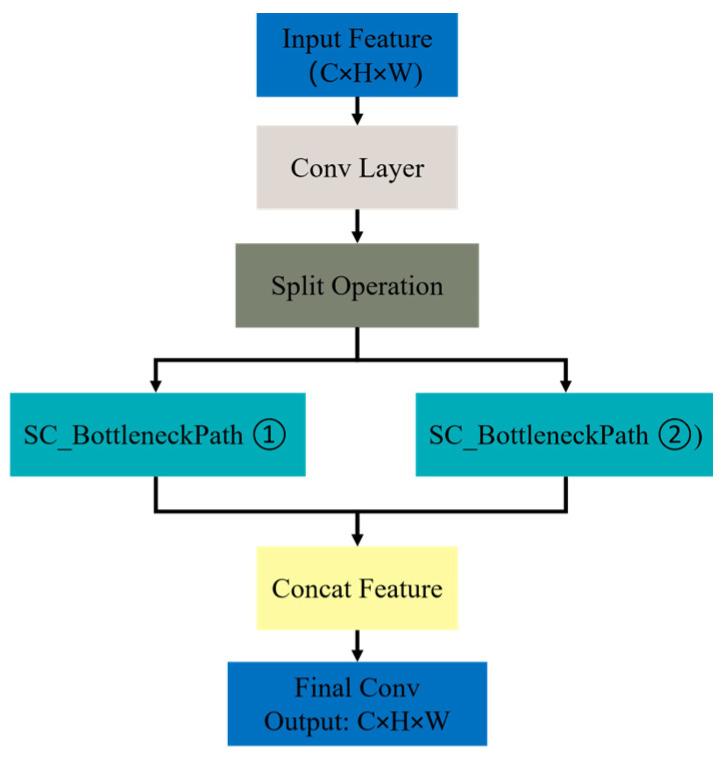
C2f-SCConv structure.

The C2f-SCConv structural module, as illustrated in Figure 7, systematically replaces all standard bottleneck modules with SC_Bottleneck path units to reconstruct the original C2f architecture, thereby profoundly optimizing the feature modeling process. This design thoroughly integrates an adaptive gradient truncation strategy with a cross-stage feature pyramid fusion mechanism. On the one hand, it improves the learning capacity of cross-level feature diversity in the spatial dimension; on the other hand, it increases the model’s processing efficiency for redundant gradient information. It effectively promotes the lightweight design of the entire network by advancing lightweighting and simultaneously compressing parameters and reducing computational complexity.

## 4. Results

### 4.1. Experimental Setting

These experiments were performed on a device running Windows 10 (Microsoft Corp., Redmond, WA, USA), with an Intel i5-13500H CPU (Intel Corp., Santa Clara, CA, USA) and an RTX 3080 TI GPU with 16GB of VRAM (NVIDIA Corp., Santa Clara, CA, USA). The software stack included PyTorch version 1.12.0 (Meta Platforms, Inc., Menlo Park, CA, USA), Torch version 1.10.2 (Meta Platforms, Inc., Menlo Park, CA, USA), and CUDA 11.3 (NVIDIA Corp., Santa Clara, CA, USA) graphics drivers.

### 4.2. Data Collection, Annotation, and Processing

**Figure 8:**
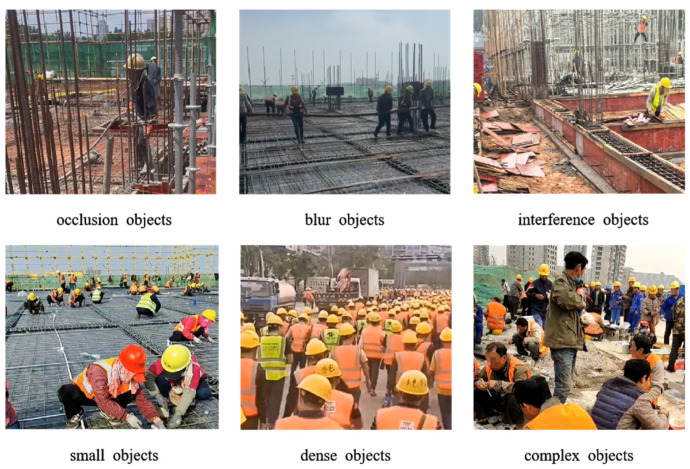
Example of specific scene images.

The experiment utilizes the open-source Safety Helmet Wearing Dataset (SHWD), which was carefully analyzed and reorganized to create the specialized Safety Helmet Compliance Testing (SHCT) dataset [29,30]. The original SHWD contains 17,839 images. Nonetheless, model overfitting can be easily triggered by factors such as the irregular distribution of label categories and the restricted number of images within the dataset. To solve this problem, we picked 10,000 great photos from SHWD and rebuilt them into the SHCT set. There are things with perspective, small things to aim at, and many close-up things in the scene, just like what you see in Figure 8.

In order to improve the accuracy of our test, we carefully annotated the SHCT pictures. We divided all the pictures into two different groups: “people” and “helmets”. We used a standard tool called LabelMe to convert the dataset into YOLO format. We divided the picture into three different parts: training, validation and testing. This arrangement can ensure that we can evaluate the data fairly, train the model systematically, and observe the effect of the model.

### 4.3. Model Testing and Evaluation Metrics

The YOLOv8n-L model was trained for 160 iterations, with each batch containing 20 images. Weight decay was set to 0.0005, and the learning rate was initialized at 0.01.

In order to see how effective this model is in identifying safety helmets, we analyzed three key indicators: Precision (precision rate), Recall (recall rate) and Average precision (mAP). These indicators are selected to comprehensively evaluate the performance of the target detection model, including accuracy and speed [31].

(1) Precision (P) measures the truly "good" proportion in the samples predicted by the model, which is used to indicate the accuracy of the model in positive prediction, as in Formula (5). TP (True Positive) is the count of good samples rightly identified as good, while FP (False Positive) is the count of bad samples wrongly called good.

(5) $$Precision=\frac{TP}{TP+FP}$$

(2) Recall (R) evaluates the model’s positive sample detection. It is the percentage of positive samples accurately anticipated as positive. See Formula (6):

(6) $$\mathrm{Recall}=\frac{TP}{TP+FN}$$

These include FN (False Negative), which indicates positive samples misclassified as negative.

(3) The mean mAP is a standard metric for identifying frequent items, which summarizes a model’s detection performance across multiple thresholds. Average Precision computes the mean mAP across all categories. AP calculates the area beneath the Precision–Recall curve to evaluate the detection performance of a specific category [32]. Formulas (7) and (8) illustrate the methodology by which mAP calculates the average of AP values across all categories:

(7) $$AP=\int_{0}^{1}P\left(R\right)dR$$

(8) $$mAP=\frac{1}{N}\sum_{j=1}^{N}AP_{j}$$

### 4.4. Ablation Experiment

We made a comparative experiment, comparing the basic YOLOv8n model with the version with new functions, and the specific results can be seen in Table 1.

A represents YOLOv8n+CA, B represents YOLOv8n+PCD, and C represents YOLOv8n+SCConv. The improved YOLOv8n model significantly improved mAP performance by introducing a coordinate attention module, as shown in Table 1, which increased from 93.9% to 95.2%. But this improvement comes at the cost of increasing the model volume by 0.41 MB and the number of parameters by 0.2 MB. This indicates that the CoordAttention module does help improve the accuracy of the algorithm, proving that it can make the network run better. Although the mAP of YOLOv8n+PCD decreased by 4.1% compared to YOLOv8n+CA, the model size, FLOP, and computational complexity were significantly reduced, which played a significant role in lightweighting the algorithm. The mAP of YOLOv8n+SCConv slightly improved compared to B and C, with no significant changes in model size, FLOP, and computational complexity. This also indicates that the SCConv module can improve the feature extraction performance of the algorithm and enhance detection performance. The improved YOLOv8n-L model showed a 0.5% increase in mAP score compared to the original YOLOv8n model, indicating that the algorithm optimized YOLOv8n-L model reduced the model size by 2.21 MB while improving accuracy. The number of parameters decreased by 36%, floating-point operations decreased by 49.9%, frame rate increased by 12.6%, and overall model volume decreased by 2.21 MB. Comparative analysis shows that YOLOv8n-L is significantly better than YOLOv8n in all indicators.

In summary, the ablation experiment demonstrated the performance of YOLOv8n, its independent modules, and the final model, providing support for the design of YOLOv8n-L.

### 4.5. Comparative Experiment

To test how well YOLOv8n-L performs, it is compared with several well-known object detection algorithms, including YOLOX, SSD, YOLOv5s, Faster R-CNN, YOLOv8n, and others [20]. Table 2 compares these algorithms in terms of model size, mAP, number of parameters, and frames per second. Experimental data indicate that YOLOv8n-L achieves a balance among accuracy, model complexity, and detection speed. MAP went up by 0.5 percentage points compared to YOLOv8n. YOLOv8n-L achieved 152 frames per second, which is significantly higher than the non-lightweight version. This shows that it can detect things in real time better.

The model size and parameters of YOLOv8n-L are smaller. To make things even better, the model size has been reduced from 5.98 MB to 3.77 MB as the computational cost of the computer has been reduced. Even if the mAP is a little less than usual, YOLOv8n-L can achieve better results with less computation. It improves the rapid detection ability without reducing the accuracy, and highlights the excellent efficiency and extremely fast processing performance of YOLOv8n-L.

**Figure 9:**
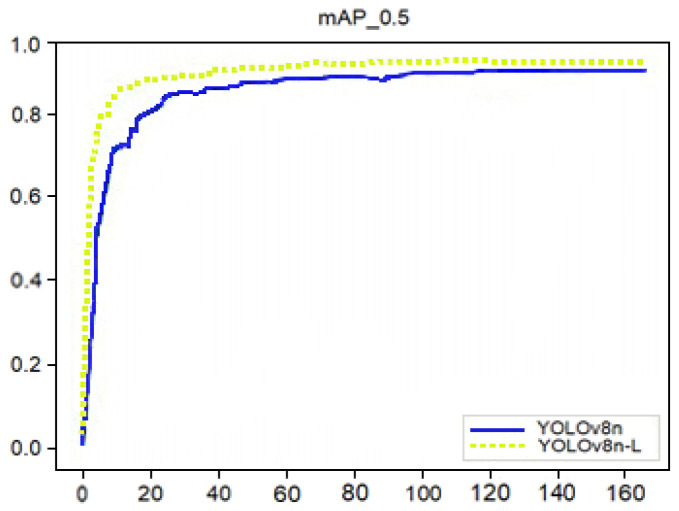
The mAP_0.5 curves of YOLOv8n-L and YOLOv8n are different.

Figure 9 shows the difference in mAP values between YOLOv8n and yolov8n-L. YOLOv8n-L realizes fast detection processing by ensuring correctness, occupying less computing resources, and being more economical. It can better combine speed with actual demand.

### 4.6. Comparative Analysis of Monitoring Effectiveness

**Figure 10:**
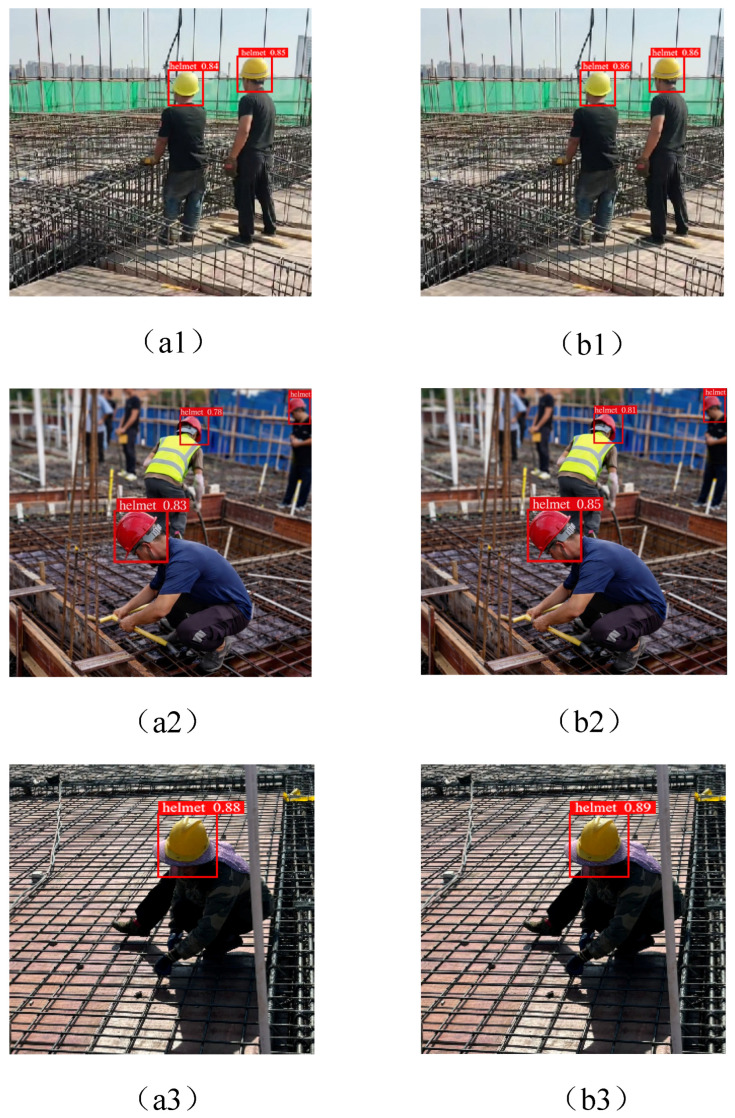
The detection performance of YOLOv8n-L and YOLOv8n. (**a1**,**b1**) show normal scenarios; (**a2**,**b2**) show occlusion scenarios; (**a3**,**b3**) represent other scenarios.

Figure 10 shows a qualitative comparison of the detection performance of our proposed YOLOv8n-L model and YOLOv8n in multiple construction site scenarios. Figure 10a1,b1 indicate that YOLOv8n-L provides more accurate and stable helmet positioning in traditional settings, while YOLOv8n occasionally generates inaccurate or incomplete bounding boxes, thereby affecting detection reliability. From a visual perspective, under normal working conditions, YOLOv8n-L has a faster response speed and higher classification accuracy.

In more complex environments, the performance gap increases, as shown in Figure 10a2,a3,b2,b3. YOLOv8n exhibits false negatives and positioning errors when encountering different perspectives, partial occlusion, and overlapping objects. YOLOv8n-L exhibits better robustness to these difficulties while maintaining precise positioning. Even if workers are partially obscured or located in cluttered steel reinforcement areas, the enhanced model can consistently detect helmets.

Overall, visual evidence suggests that YOLOv8n-L achieves a better balance between robustness, inference efficiency, and detection accuracy. In complex construction site safety monitoring scenarios, reliability under occlusion and viewpoint changes is crucial, and its improved perception ability has proven to be particularly beneficial.

## 5. Limitations and Future Work

The initial version of the YOLOv8n model faces challenges in file size and deployment cost, which affect its practicality. Our proposed method effectively alleviates these limitations, improves the performance of field application, and can realize rapid detection and deal with complex environments. The tests conducted indicate that YOLOv8n-L makes finding things immediately better by making the model smaller and using less computer power without losing its correctness. YOLOv8n-L is a lightweight approach to YOLOv8n that balances speed, accuracy, and computer power usage, making it suitable for fast helmet detection and using fewer computing resources.

The ongoing work has two limitations: Firstly, although the dataset that can be used is reliable, its small size and limited scene types make the model unable to work properly under different lighting conditions, hiding some things in certain scenes and from many light points. Second, a lightweight design enables accuracy optimization [33]. Based on this, future work will proceed in the following directions: First, constructing multimodal and multi-perspective construction scene datasets, and introducing synthetic data augmentation techniques such as GANs, NeRF, and domain randomization to significantly enhance the model’s adaptability in complex scenarios involving lighting variations, occlusions, and multiple angles. Second, exploring neural architecture search (NAS) to automatically discover more efficient lightweight network structures, breaking through current accuracy bottlenecks while maintaining compact model size. Third, conducting model quantization research, particularly INT8 quantization, to reduce deployment costs on edge devices and further enhance inference speed and energy efficiency. Fourth, introducing knowledge distillation strategies to construct ultra-lightweight student models, enabling high-precision detection solutions better suited for embedded scenarios. Overall, future research will focus on achieving a better balance between lightweight design and high accuracy while enhancing model robustness across diverse construction environments, thereby supporting safer and more reliable intelligent on-site monitoring systems.

## 6. Conclusions

Manual inspection methods require time and labor and are susceptible to weather and human factors. Current safety helmet identification algorithms have achieved notable accuracy; however, two fundamental concerns remain. First, this approach is challenging to apply to resource-constrained applications due to the significant rise in model parameters and computational resource requirements. Second, complicated backgrounds, occlusions, and small targets at construction sites make it difficult to increase detection accuracy. Due to these challenges, we chose YOLOv8n as the baseline model and concentrated on detection accuracy and model lightweighting. Experimental results show that the modified YOLOv8n-L improves detection accuracy. The first version of YOLOv8n-L had a high cost, required a large amount of calculations and parameters, and was not suitable for on-site application. The advantage of low-cost deployment of YOLOv8n-L allows us to use it more easily, which makes the algorithm more efficient. The experimental results showed that the mAP of lightweight YOLOv8n-L was 94.4%. The new model is 2.21 M smaller and has 1.09 M fewer parameters than the first YOLOv8n model. Therefore, finding the safety helmet is now easier and more accurate. The YOLOv8n-L method for finding safety helmets makes the model smaller, reduces the number of parameters, makes calculations easier, and still enables accurate detection.

## Figures and Tables

**Table 1:** Ablation experiment comparison.

Method	mAP/%	FLOPS/G	Params/M	Model Size/MB	P/%	R/%
YOLOv8n	93.9	8.3	3.03	5.98	91.6	86.8
A	95.2	8.3	3.23	6.39	92.4	87.8
B	94.1	4.1	1.98	3.81	91.8	87.5
C	95.3	8.1	3.2	6.02	92.8	87.6
YOLOv8n-L	94.4	4.2	1.94	3.77	92.2	87.4

**Table 2:** Results of the experiment.

Method	mAP_0.5/%	FPS	Params/M	Model Size/MB
YOLOX	93.2	110	16.4	16.2
SSD	83.6	92	97.7	108.2
YOLOv5s	91.2	121	14.5	14.12
Faster-RCNN	90.8	85	106	184.8
YOLOv8	93.1	128	16	7.21
YOLOv8n	93.9	135	3.03	5.98
YOLOv8s	93.5	139	12.7	7.27
YOLOv8n-L	94.4	152	1.94	3.77
YOLOv11	96.3	208	2.98	5.08

## Data Availability

The data supporting the findings of this study are available from the first author, Xinyu Zuo, upon reasonable request.
